# Theory and Practice

**Published:** 1863-03

**Authors:** W. H. Atkinson


### THEORY AND PRACTICE.

Read before the New York Society of Dental Surgeons, September 17, 1862.

BY W. H. ATKINSON.

To the confined limits of practical demonstration more than to the wildest theoretic fancy are we indebted for our *stay* in low developments. Each lilliputian who prides himself upon his *practical* rather than his theoretic "book knowledge," as he denominates all search for the grand thorough-base of *principle*, looks with pity or astonishment, one or both, upon the earnest searcher after the types of genera and species of bodies and principles in small and great things alike; wondering what interest there possibly can attach to the mere speck of an organic structure which is handled with such anxious care in preparing it for microscopic inspection, and the patient survey of immense, unwieldy masses, without difference enough to make a note of on the other, while all the advantages of ordinary amusement and "polished society" are foregone for the sake of this isolated labor! Alas for lilliput; he has never heard the still small voice of the angels of a truly refined society encouraging him to do and communicate good in preference to the more selfish exercise of getting good only. Had his vision once have been truly opened to the grand schemes of life and being, that patiently await apprehension and promulgation to all in need of clearly defined and useful knowledge, *he* too could forget his meals and courted pillow, for the glory of tracing how exactly the highest are indicated in the lowest, and how precisely repeated are the very lowest in the highest and completest organisms within the range of intensest vision. First forms of animal life—the protozoa—mere drops of amorphous jelly enclosed within a structureless film of the same chaos of matter, eat without mouths, digest without digesters, move without locomotive apparatus, circulate fluids without even the vestige of a vascular system, and perform all the functions of the sense of feeling without the ordinary tools of this function; in a word live, move, and have individualized being without differentiation of function or parts, being in turn all nerve, all muscle, all stomach, all heart, etc., etc., to the end of the chapter; one per cent. of which, had it found lodgment in the perception, would have riveted the attention and won the interest of this primal as well as the greatest philosopher. Why then are not all thus intensified in matter so vital to the well being of the whole race?

Because low, selfish and narrow purposes of life have supplanted the sense of high mission for good to somebody to be performed by every capable being upon the planet! The anxieties of pupils are more directed to the how than the why of life and being! What will such and such authorities approve or disapprove is with most a much more stressful query than what will my own highest intelligence unbiasedly dictate to be done!!

He who is not enthused with a high and holy purpose has no business to obtrude himself into any profession! Professions were established for the amelioration of a state of deficiency into which the race by some means had found itself, and he who has no knowledge of the former state of wholeness can have no just power of discrimination as to when it is attained; so he who has no well defined theory can have no settled, regular and certain method of practice in any case: and hence is but an experimentor.

This thing of making bold or timid strokes in the dark, and trusting to the blind chance of luck to come out successfully, has held the dominion among us quite too long already, and it is high time that teacher and pupil both come frankly to the acknowledgment.

"That all there is of e-du ca-tion is the solution of the difficulties which present themselves to us in study and in practice," and the sooner the starched professorships and false dignities are completely washed away by the solvent power of vigorous questioning of all and of each, the shorter will be the period of our metamorphosis out of the present larval state of our dental schools and private tutorage!

If the question were put to-day to every pupil of merit in the schools or offices, and every member of every faculty, and each who have been engaged to teach a pupil "what he could,"—"Who has been most benefitted by the contact of teachers and learners?" How many think you have paid sufficiently close attention to their progresses to entitle them to the appellation of competent witnesses? Doubtless the great majority of pupils would frankly acknowledge that they had received much more than they had given, but that professor and masters would unhesitatingly endorse the statement, I more than doubt! (if they bore true testimony.)

If we will hold every man who pretends to know, or who looks as if he ought to know, any thing of interest to the profession and the world, up to such close scrutiny of interrogative examination that he will be forced to let the light that is in him shine out, or prove it to be but the corruscations of the "ignis fatuus" of dark ignorance and self-satisfied assumption. For far better is it for him and for all that he be made to illuminate or be illuminated for future usefulness, or driven from his position as a false light not worthy our worship, confidence, or the space occupied to the exclusion of that which might be proper, progressive and principal! Complete junction of correct knowledge of minutest part and most extended relation can alone be guide to certitude and safe interference and management of the living economy in states of health or disease.

So little have theories been understood and taught, that there are scarcely two practitioners capable of comprehending each other's theories, when purely original in announcement, and at all reconciling them to any practice based upon intelligent knowledge of structures and remedies! The only way that this can be accounted for is to suppose that theory is for the "books," the literature of the profession, and that practice is for the patient or for the pocket.

Such execrable theory and such damaging practice as finds its way into the records of the profession are enough to make an esculapian of the dark ages blush to his most distant capillary. Sometimes when our heads are as wrong as our left hands, our surroundings will awake enough earnestness in our hearts to overbear all our bad precedents of false doctrine, and inspire us for the time with a will and a purpose which result in a satisfactory service to him who happens to be in the chair for the time. But if we are coarse grained and set in former errors, this will be the exceptional condition, and our general practice will gravitate to the standard of our crude conceptions and stubborn theories, which are apt to be followed by men of a certain age, despite the daily testimony of cases which ought to overthrow and correct such doctrines of darkness, imbibed in a time when there was more excuse to blunder than now falls to the lot of him who has his eyes open, his head sober, and his great palpitating heart in the right place; whether his hand has emerged from the education of its sense of touch, concomitant with the stiff, coarse and ponderous instruments of the past, or not.

It is not every practitioner whose head and purpose have become enlightened and advanced, that can bring up his hand to the delicate dexterity of a really fine and magnificent work, such as are many of our most difficult operations now called for by a few intelligent patients.

